# Comparison of interrupted and continuous modulator therapy in cystic fibrosis: a real life experience in Turkey

**DOI:** 10.3389/fped.2025.1659633

**Published:** 2025-10-13

**Authors:** Almala Pinar Ergenekon, Merve Selcuk, Gokcen Unal, Gamzegul Gozen Bayramoğlu, Cansu Altuntas, Mehmet Kose, Abdurrahman Erdem Basaran, Sinem Can Oksay, Salih Uytun, Sedat Oktem, Zeynep Seda Uyan, Yakup Canitez, Esen Demir, Velat Sen, Veysel Karakulak, Ela Erdem Eralp, Bulent Karadag, Fazilet Karakoc, Sevgi Pekcan, Tugba Sismanlar Eyuboğlu, Erkan Cakır, Yasemin Gokdemir

**Affiliations:** ^1^ Division of Pediatric Pulmonology, Marmara University School of Medicine, Istanbul, Türkiye; ^2^ Division of Pediatric Pulmonology, Necmettin Erbakan University, Konya, Türkiye; ^3^ Division of Pediatric Pulmonology, Gazi University, Ankara, Türkiye; ^4^ Division of Pediatric Gastroenterology, Istinye University, Istanbul, Türkiye; ^5^ Division of Pediatric Pulmonology, Erciyes University, Kayseri, Türkiye; ^6^ Division of Pediatric Pulmonology, Akdeniz University, Antalya, Türkiye; ^7^ Division of Pediatric Pulmonology, Medeniyet University, Istanbul, Türkiye; ^8^ Division of Pediatric Pulmonology, Ankara Bilkent Şehir Hastanesi, Ankara, Türkiye; ^9^ Division of Pediatric Pulmonology, Medipol University, Istanbul, Türkiye; ^10^ Division of Pediatric Pulmonology, Koc University, Istanbul, Türkiye; ^11^ Division of Pediatric Pulmonology, Bursa Uludag University, Bursa, Türkiye; ^12^ Division of Pediatric Pulmonology, Ege University, Izmir, Türkiye; ^13^ Division of Pediatric Pulmonology, Dicle University, Diyarbakır, Türkiye; ^14^ Division of Pediatric Allergy, Cukurova University, Adana, Türkiye; ^15^ Division of Pediatric Pulmonology, Istinye University, Istanbul, Türkiye

**Keywords:** interrupted modulator therapy in cystic fibrosis, reimbursement of modulator therapy in cystic fibrosis, pulmonary function test, body mass index, CFTR modulator drugs

## Abstract

**Introduction:**

Cystic fibrosis (CF) transmembrane conductance regulator protein (CFTR) modulators have significantly improved health outcomes in patients with cystic fibrosis (pwCF). However, in Turkey, access is limited due to lack of insurance coverage, and treatment is only granted in 3-month periods via court rulings. This study aimed to compare clinical outcomes between patients receiving continuous vs. intermittent modulator therapy.

**Methods:**

In this retrospective multicenter study, data from 229 CF patients across 14 centers in Turkey who received highly effective modulator therapy (HEMT) for at least six months were analyzed. Patients were grouped based on whether they received treatment continuously (Group 1) or with interruptions (Group 2). Changes in percent predicted forced expiratory volume in one second (ppFEV₁) and body mass index (BMI) were evaluated at baseline, 3 months, and 6 months. For Group 2, ppFEV₁ was also assessed during interruption periods.

**Results:**

Of the 229 patients, 38.4% received continuous treatment while 61.5% experienced treatment interruptions. Both groups showed significant improvements in ppFEV₁ over six months (*p* < 0.001). However, Group 2 experienced a significant decline during interruption periods (*p* < 0.001), followed by recovery upon reinitiation. BMI also increased significantly in both groups (*p* < 0.05). Patients with baseline ppFEV₁ < 70% showed greater improvement compared to those with milder disease.

**Conclusion:**

Short-term clinical outcomes in ppFEV₁ and BMI were similar between continuous and intermittent treatment. However, treatment interruptions may reduce cumulative benefits, potentially impacting long-term outcomes. Ensuring uninterrupted access to HEMT is essential, especially in low- and middle-income countries**.**

## Introduction

Cystic fibrosis (CF) is a severe autosomal recessive ion channel disorder. Since its clinical description over 80 years ago, the majority of treatments have focused on alleviating symptoms caused by dysfunction of the cystic fibrosis transmembrane conductance regulator (CFTR) protein (1). Of the more than 2,100 identified variants in the *CFTR* gene, over 1,000 are associated with CFTR dysfunction, which is the hallmark of cystic fibrosis. The recent introduction of highly effective CFTR modulators has led to substantial health improvements, with approximately 90% of CF patients being eligible based on their CFTR mutations (2, 3).

CFTR modulators have demonstrated numerous benefits, including increased weight and body mass index (BMI), reduced sweat chloride concentrations, improved quality of life and percent predicted forced expiratory volume in one second (ppFEV₁), and decreased pulmonary exacerbations (4–7). Furthermore, interim results from a long-term registry-based study showed a 72% reduction in mortality and an 85% reduction in lung transplantation rates compared to the year before the availability of the therapy Elexacaftor/Tezacaftor/Ivacaftor (ELX/TEZ/IVA) (8). A recent open-label study in children aged 2–5 years also confirmed that ELX/TEZ/IVA is generally safe and well-tolerated, consistent with findings in older age groups (9).

Although these modulators are available in many countries, access remains limited in low- and middle-income countries due to their high cost, leading to significant disparities in CF outcomes. In Turkey, CFTR modulators are not reimbursed by the national healthcare system. Access depends on court rulings. People with cystic fibrosis (pwCF) who carry at least one copy of the F508del mutation in the cystic fibrosis transmembrane conductance regulator (CFTR) gene, or another mutation documented as responsive to ETI, receive ETI therapy. Similarly, pwCF who carry at least one CFTR mutation documented as responsive to IVA receive IVA therapy (10). There are no additional country-specific restrictions or differences in variant eligibility in Turkey. However, after court decision, drugs are provided for only 3-month periods. During gaps between court decisions, many patients experience interruptions in treatment, and only a small proportion of patients can maintain continuous therapy. Limited data exist on the clinical consequences of intermittent CFTR modulator use, particularly in children (7, 11).

In this study, we aimed to summarize our experience with intermittent modulation therapy and to compare the clinical characteristics and outcomes of individuals who received intermittent vs. continuous treatment.

## Methods

This retrospective study included patients receiving CFTR modulator therapy from 14 centers in Turkey between June 2021 and June 2024. The diagnosis of CF was established based on CFTR gene sequence analysis and multiplex ligation-dependent probe amplification (MLPA), sweat test results, and clinical status. Inclusion required the presence of CF-causing mutations confirmed by genetic testing, and all enrolled patients fulfilled this criterion. Since neonatal screening for CF has been available in Turkey since 2015, younger patients were predominantly diagnosed through the national screening program, whereas older patients were identified according to clinical manifestations.

Patients were included if they had received highly effective modulator therapy (HEMT) for a minimum of 6 months. In our cohort, individuals carrying class 2 mutations (such as F508del in homozygosity or heterozygosity), as well as those with other CFTR variants eligible for therapy, received triple therapy with elexacaftor/tezacaftor/ivacaftor (ETI). Patients with class 3 gating mutations, as well as class 4 and class 5 mutations defined as responsive, were treated with Ivacaftor monotherapy.

Data were collected on demographic characteristics, sweat chloride test values, ppFEV₁, BMI, and any potential adverse effects of treatment at baseline, during treatment, and after treatment interruption. Changes (*Δ*) in ppFEV₁ and BMI were defined as percentage differences relative to baseline values.

Patients were categorized into two groups: Group 1 included those who continued treatment without any interruption, and Group 2 included those who experienced a treatment cessation period. An interruption was defined as a gap of at least two weeks following a 3-month treatment course granted via court decision, before the next treatment course began.

In patients receiving CFTR modulators, sweat testing using the chloride level method was routinely performed at baseline and at the 3rd month of therapy.

All spirometric tests were performed uniformly across all centers in accordance with ATS/ERS guidelines (12). For both groups, ppFEV₁ values were recorded at baseline, 3 months, and 6 months after initiating HEMT. In a subgroup of patients receiving intermittent therapy, ppFEV₁ measurements were also assessed at the end of the cessation period and immediately before the resumption of therapy. Lung disease severity was classified based on baseline ppFEV₁: 70%–90% was considered mild, 40%–69% moderate, and <40% severe (13).

BMI values for individuals with CF under 18 years of age were calculated using BMI percentile charts (z score), whereas for those over 18 years of age, BMI was calculated. BMI and BMI z score were measured at baseline and at 6 months in both groups.

Patients' colonization status with Pseudomonas aeruginosa (PsA) or Methicillin-Resistant Staphylococcus Aureus (MRSA) were recorded at baseline. Samples were obtained as sputum from patients able to expectorate, and by cough swab from those who could not. Patients in both groups were also stratified by the presence of chronic PsA infection. Chronic PsA infection was defined as having positive PsA cultures in at least 50% of the months over a one-year period in which samples were collected (14).

Data analysis was performed using the Statistical Package for the Social Sciences (SPSS, version 23.0). Descriptive statistics included frequencies, means, standard deviations (SD), and interquartile ranges (IQR), depending on the distribution of continuous variables. For comparisons of nonparametric continuous variables between two groups, the Mann–Whitney U test was used. The Friedman test was applied for repeated measures across more than two dependent time points, with Bonferroni correction applied for multiple comparisons (*p* < 0.005/n). A *p*-value < 0.017 (0.05/3) was considered statistically significant when comparing three time points. Chi-square or Fisher's exact tests were used to compare categorical variables between groups. A *p*-value < 0.05 was considered significant for comparisons between two groups. Spearman's rank correlation coefficient (*ρ*) was used to assess correlations between 6-month changes in ppFEV₁ and variables such as age, age at diagnosis, BMI, and the duration of treatment interruption.

The study and protocol were reviewed by Ethical Committee of Marmara University Medical Faculty (Approval number: 09.2024.780). Written consent was obtained from both the participants and their families.

## Results

A total of 229 people with cystic fibrosis (pwCF) were included in the study, of whom 105 were aged ≥18 years. The median age was 16.1 years (IQR: 10.2–22.7), and the median age at diagnosis was 4.0 months (IQR: 2.0–42.0). Among the participants, 88 (38.4%) received highly effective modulator therapy (HEMT) continuously (Group 1), while 141 (61.5%) experienced treatment interruption (Group 2). The demographic and clinical characteristics of the study population are summarized in Table 1. In Group 2, the median total duration of treatment interruption was 60 days (IQR: 30.0–150.0).

**Table 1:** Demographic and characteristic features of the study population.

Characteristics	Group 1 (continuous use) *N* = 88 (38.4%)	Group 2 (intermittent use) *N* = 141 (61.5%)	*P* value
Sex, Female, *n* (%), 112 (48.9%)	38 (43.2%)	74 (52.5%)	0.20
Age (year), median (IQR)	13.26 (8.45–21.79)	18.10 (11.13–23.06)	***0***.***02***
Age at diagnosis (month), median (IQR)	4.00 (1.00–24.0)	4.00 (2.00–60.00)	0.34
Number of pwCF, ≥ 18 years of age, *n* = 105	33 (37.5%)	72 (51.1%)	0.58
Gene variant, n(%) (Ivacaftor)			0.75
Class 3 gating mutations			
R347H	-	1 (2.7%)	
G178R	-	1 (2.7%)	
Class 4 mutations	4 (10.8%)	7 (18.9%)	
Class 5 mutations	8 (21.6%)	13 (35.1%)	
Unclassified	1 (2.7%)	2 (5.4%)	
Gene variant, n(%) (Elexacaftor/Tezacaftor/Ivacaftor)			0.34
F508del			
Homozygous	16 (8.3%)	47 (24.5%)	
Heterozygous	42 (21.9%)	51 (26.6%)	
Other mutations	17 (8.8%)	19 (9.9%)	
ppFEV1, median (IQR), *n* = 215	81.0 (53.2–94.0), *n* = 84	67.0 (42.0–94.0), *n* = 131	0.10
BMI, median (IQR)			
< 18 years of age, z score	−0.77 (−1.71 to 0.19)	−0.72 (−1.25 to −0.20)	0.81
≥ 18 years of age	18.80 (16.49–22.49)	19.62 (18.01–21.90)	0.26
Colonization, *n* (%)			
PsA	29 (33.7%)	67 (47.5%)	***0***.***04***
MRSA	4 (4.2%)	20 (14.2%)	***0***.***04***
Pankreatic insufficiency, *n* (%)	71 (82.6%)	119 (84.4%)	0.85
ABPA, *n* (%)	2 (2.4%)	4 (2.84%)	1.00
CFLD, *n* (%)	3 (3.5%)	10 (7.1%)	0.38
CFRD, *n* (%)	5 (5.8%)	14 (9.9%)	0.40
NIPPV, *n* (%)	1 (1.4%)	9 (7.4%)	0.09
Oxygen support, *n* (%)	2 (2.9%)	15 (12.0%)	0.05
Age at initiation of therapy, median (IQR), year	12.16 (7.66–20.05)	15.96 (9.66–20.70)	0.07

ABPA, allergic bronchopulmonary aspergillosis; BMI, body mass index; CF, cystic fibrosis; CFRD, CF-related diabetes; CFLD, CF-related liver disease; ppFEV, forced expiratory volume in 1 s; IQR, interquartile range; HEMT: highly effective modulator treatment; MSSA, *Methicillin-Sensitive Staphylococcus Aureus*; MRSA, *Methicillin-Resistant Staphylococcus Aureus*; NIPPV, non-invasive positive pressure ventilation; Pred, predicted; PsA, *Pseudomonas Aeruginosa*.

Values in bold indicate statistical significance (*P* < 0.05).

### ppFEV₁ status

In Group 1, among 54 pwCF with repeated measurements, median ppFEV₁ increased from 81.00 (IQR: 52.75–93.25) at baseline to 90.50 (71.50–101.50) at 3 months and 94.00 (79.25–102.00) at 6 months, with a statistically significant improvement (*p* < 0.001, 95% CI: 0.000–0.054).

**Figure 1:**
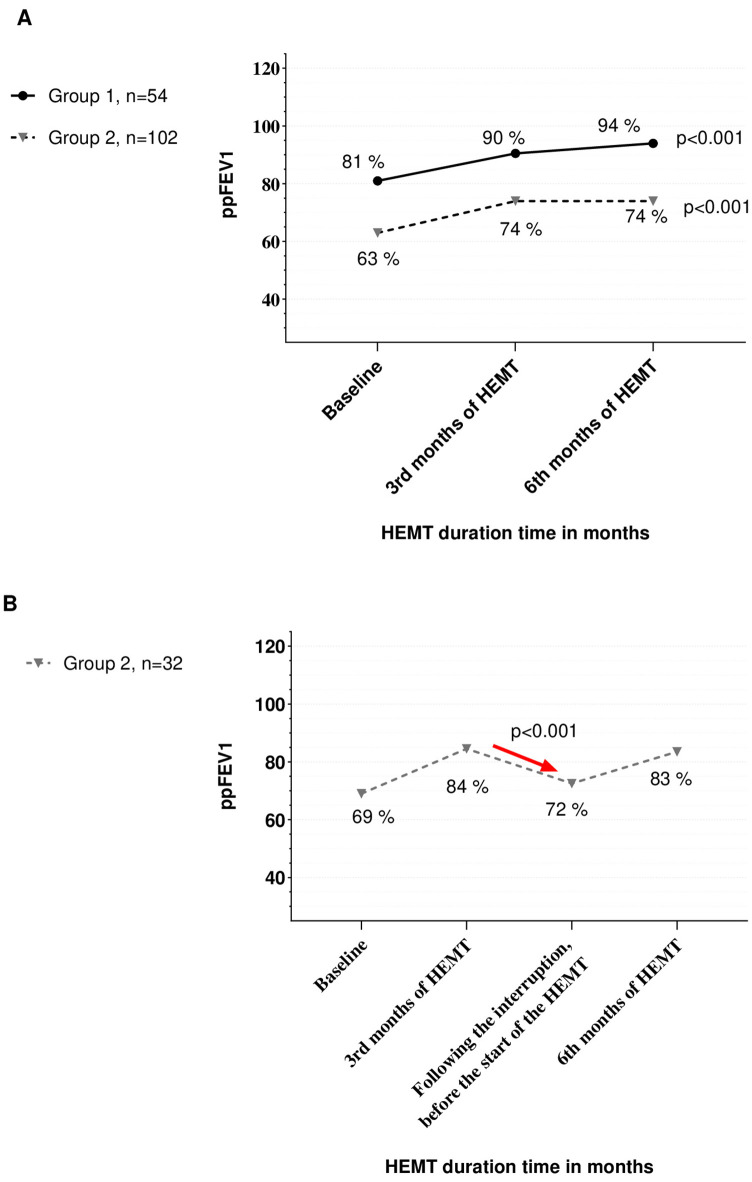
**(A)** The ppFEV_1_ values over 6 months of the HEMT in both groups. **(B)** The ppFEV_1_ values over 6 months of the HEMT in Group 2.

In Group 2, among 102 pwCF with repeated measurements, median ppFEV₁ increased from 63.00 (40.00–91.00) at baseline to 74.00 (50.75–101.00) at 3 months and 74.00 (49.00–100.25) at 6 months (*p* < 0.001, 95% CI: 0.000–0.029) (Figure 1A).

There was no significant difference in the 6-month percentage increase in ppFEV₁ (*Δ*FEV₁) between the groups: median *Δ*FEV₁ was 14.0 (4.9–28.5) in Group 1 and 14.3 (3.1–28.3) in Group 2 (*p* = 0.616). Furthermore, *Δ*FEV₁ was not correlated with the number of days of HEMT interruption in Group 2 (r = −0.09, *p* = 0.41).

In Group 2, only 32 patients had pulmonary function tests available just before reinitiating HEMT. These patients did not differ significantly from the rest of Group 2 in terms of baseline ppFEV₁, BMI, or demographics. In this subgroup, median ppFEV₁ increased from 69.00 (42.50–87.25) at baseline to 84.50 (48.50–102.25) at 3 months, decreased to 72.50 (43.75–85.50) after treatment interruption, and increased again to 83.50 (48.25–101.50) at 6 months. Both the decline after interruption and the improvement by 6 months were statistically significant (*p* < 0.001 for both) (Figure 1B).

Neither chronic *Pseudomonas aeruginosa* (PsA) infection, gender, nor the presence of the F508del mutation had a significant effect on *Δ*FEV₁ at 6 months in either group.

**Figure 2:**
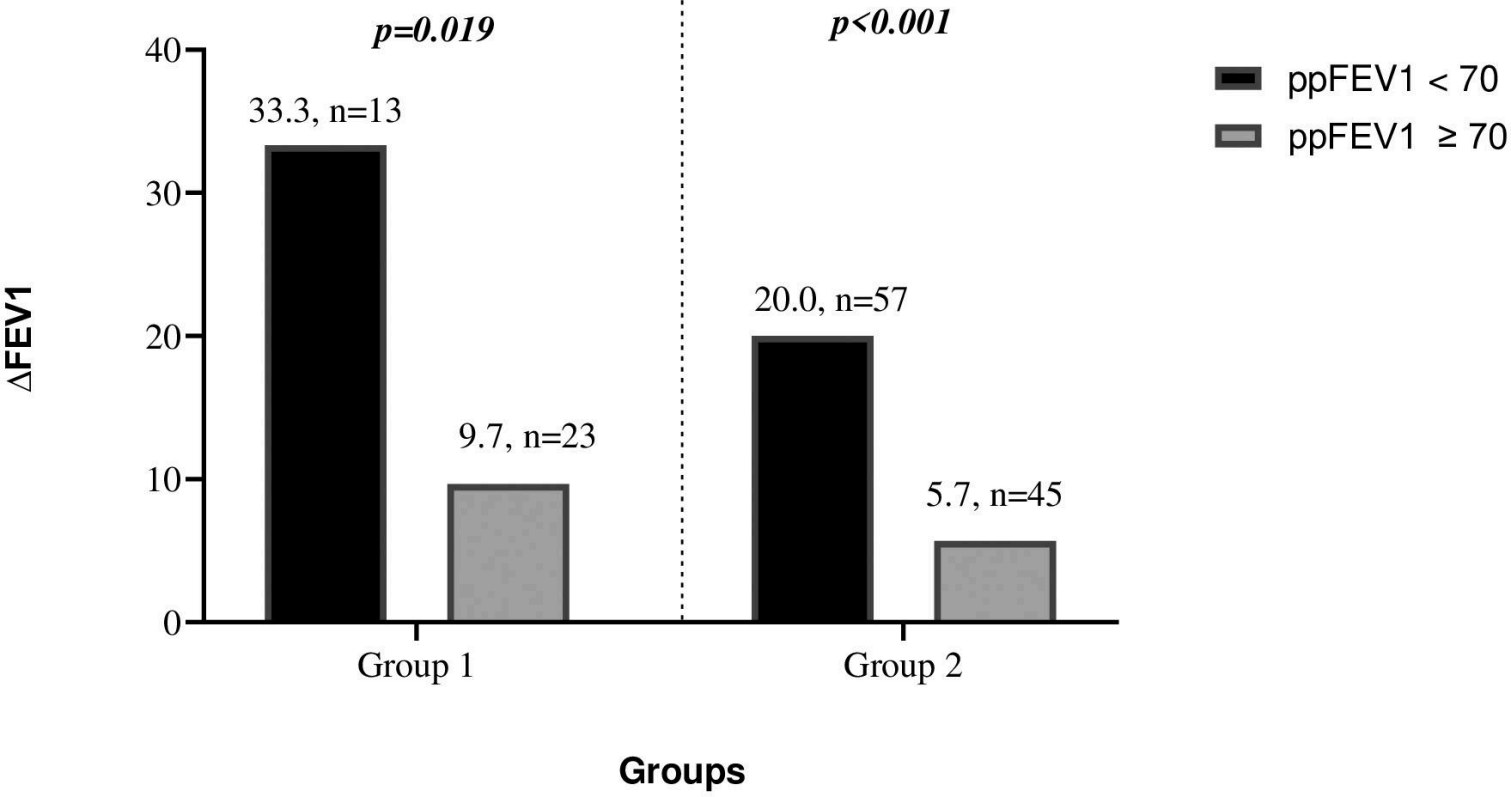
The 6-month *Δ*FEV1 changes in patients with ppFEV1 values below and above 70% in both groups.

Patients were stratified by baseline ppFEV₁ values into two categories: < 70% and ≥70%. In both groups, *Δ*FEV₁ was significantly higher among patients with baseline ppFEV₁ < 70% compared to those with ≥70% (Group 1: *p* = 0.019; Group 2: *p* < 0.001) (Figure 2). However, there was no statistically significant difference in *Δ*FEV₁ between Group 1 and Group 2 at 6 months (*p* > 0.05). Similarly, PsA infection status did not significantly affect *Δ*FEV₁ values (*p* > 0.05).

When stratified further by ppFEV₁ < 40% and ≥40%, the increase in *Δ*FEV₁ was not statistically significant in either Group 1 or Group 2 (*p* = 0.07; *p* = 0.44, respectively).

### BMI and nutritional status

**Figure 3:**
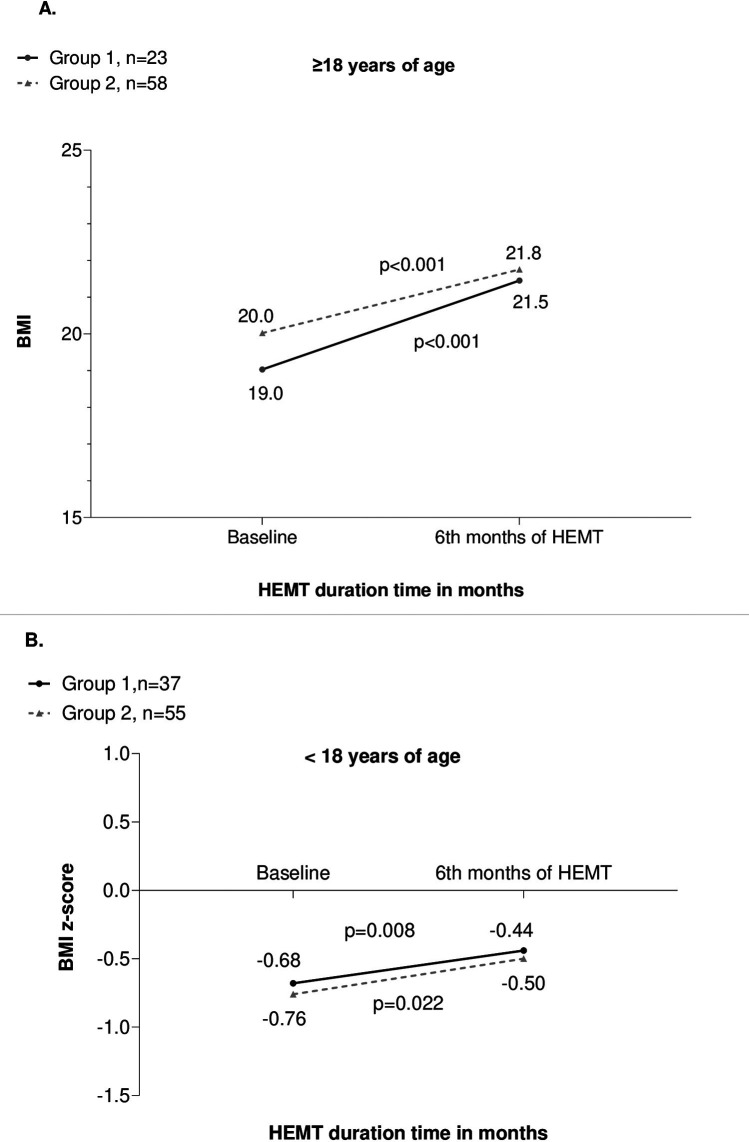
**(A)** BMI values in adults (≥18 years) over 6 months of HEMT in both groups. **(B)** BMI z-scores in children (<18 years) over 6 month of HEMT in both groups.

In patients ≥18 years old with repeated BMI measurements:

• Group 1: median BMI increased from 19.0 (18.3–22.5) at baseline to 21.5 (19.4–25.9) at 6 months (*n* = 23, *p* < 0.001, 95% CI: 0.000–0.122).
• Group 2: median BMI increased from 20.0 (18.5–22.8) to 21.8 (19.6–24.1) (*n* = 58, *p* < 0.001, 95% CI: 0.000–0.050) (Figure 3A).

There was no correlation between *Δ*BMI and the number of days of HEMT interruption in Group 2 (*r* = −0.160, *p* = 0.259).

In patients <18 years old with repeated BMI z-scores:

• Group 1: median z-score increased from −0.68 (–1.72 to 0.65) to −0.44 (–1.40 to 0.51) at 6 months (*n* = 37, *p* = 0.008, 95% CI: 0.054–0.127).
• Group 2: median z-score increased from −0.76 (–1.38 to −0.22) to −0.50 (–1.09 to 0.01) (*n* = 55, *p* = 0.022, 95% CI: 0.000–0.115) (Figure 3B).

*Δ*BMI was not correlated with the number of HEMT interruption days (*r* = 0.24, *p* = 0.877).

At the 6-month follow-up:

• In patients ≥18 years, *Δ*BMI did not significantly differ between Group 1 and Group 2 (10.63 [0.54–15.38] vs. 4.8 [–0.12–12.52]; *p* = 0.12).
• In patients <18 years, *Δ*BMI z-score also did not differ significantly between the groups (34.55 [–0.60 to 92.22] vs. 27.69 [–37.17 to 72.35]; *p* = 0.30).

Neither F508del mutation status nor gender affected *Δ*BMI at 6 months in either group.

### Sweat test and adverse effects

Sweat chloride testing was performed in 169 patients using the chloride concentration method. Sweat chloride values significantly decreased from baseline to the 3rd month of treatment in both groups. In Group 1 (*n* = 61), median values declined from 83.0 mmol/L (IQR: 76.0–93.0) at baseline to 49.0 mmol/L (IQR: 36.0–66.9) at month 3 (*p* < 0.001). Similarly, in Group 2 (*n* = 108), values decreased from 83.5 mmol/L (IQR: 69.0–94.7) at baseline to 44.0 mmol/L (IQR: 29.0–59.7) at month 3 (*p* < 0.001).

No adverse effects were observed in patients receiving Ivacaftor, whereas 30 (13.1%) patients treated with ETI experienced adverse events. The most frequently reported were rash (*n* = 12, 6.2%), abdominal pain (*n* = 3, 1.6%), and elevated liver enzymes (*n* = 3, 1.6%).

## Discussion

In this study, we observed that ppFEV1 values, BMI, and BMI z-scores improved after six months of HEMT in both the continuously treated group and the intermittently treated group. Among patients receiving intermittent therapy, ppFEV_1_ values significantly declined during treatment interruption, returning to pre-interruption levels after therapy was reinitiated. In both groups, the 6th-month ppFEV_1_ improvement was better in patients with moderate/severe lung disease than in those with mild disease. While several clinical trials and observational studies have reported improvements in various clinical outcomes following the initiation of modulator treatment, data on patients experiencing treatment interruptions are limited (11). To our knowledge, this is the first multicenter study comparing the characteristics and outcomes of patients receiving continuous vs. intermittent modulator therapy.

Previous studies have highlighted significant increases in ppFEV1 among patients treated with HEMT (5, 15, 16). Similarly, our findings revealed an initial increase in ppFEV1 within the first three months in both groups, which persisted in patients receiving continuous therapy but declined significantly in those with treatment interruptions. This pattern is consistent with a recent Turkish study, in which ppFEV1 improved during modulator therapy but dropped after discontinuation, with 66.6% of patients experiencing a decline greater than 10% (10). Additionally, Oztosun et al. reported that interrupted modulator therapy in children with cystic fibrosis was associated with declines in lung function, underscoring the importance of uninterrupted access to HEMT (17). Our results reinforce these findings, demonstrating the detrimental impact of therapy discontinuation on lung function.

Optimal nutritional care remains a cornerstone of cystic fibrosis management and is critical to improving clinical outcomes and extending life expectancy (18). Several studies have established a link between improved nutritional status and enhanced pulmonary function (19, 20). At our center, the implementation of a standardized nutritional protocol for children with CF resulted in significant increases in both BMI and ppFEV_1_ (21). HEMT has been shown to facilitate weight gain and BMI improvement through multifactorial mechanisms (5, 16, 22). In our study, BMI values improved significantly at the 6th month in both the continuous and intermittent treatment groups, consistent with previous research from Turkey (11).

Despite these improvements, our patients showed comparatively lower nutritional and pulmonary status compared to those in Europe and the United States**,** as indicated by the ECFS 2023 registry (23). In our cohort, individuals with moderate to severe lung disease exhibited more substantial gains in ppFEV_1_ at six months compared to those with mild disease. Although this trend was more prominent in the continuously treated group, the small sample size limited the ability to demonstrate statistical significance. A recent study, projecting the impact of delayed access to ELX/TEZ/IVA for pwCF revealed that earlier introduction of ELX/TEZ/IVA could reduce deaths and improve the median age of survival (24). Given the cumulative nature of therapeutic benefits, intermittent access to HEMT may result in diminished long-term outcomes, including potential declines in lung function.

In Group 2, which received interrupted therapy, patients were older, and as expected, the prevalence of PsA and MRSA colonization was higher; however, lung function parameters were not significantly lower compared to Group 1. As this is a real-life, multicenter study, patient selection was not controlled, and randomization was not possible. Since our follow-up period was less than 12 months, we were unable to evaluate the impact of ETI on PsA and MRSA colonization, as assessing colonization status reliably requires at least a 12-month observation period. Consequently, direct comparisons with studies such as Sunman et al., which reported a decrease in the prevalence of PsA and MRSA colonization following one year ELX/TEZ/IVA treatment, are not possible in our cohort (25).

According to the ECFS 2023 registry, approximately 35% of patients in Turkey have access to modulator therapy, often only through court orders, as these medications are not reimbursed by the national healthcare system (23). Treatment interruptions due to legal and financial barriers provide a unique opportunity to examine the real-world consequences of intermittent HEMT use. These findings are also relevant in countries where reimbursement exists, as issues like treatment adherence and insurance coverage may similarly lead to interruptions.

This study has several limitations. First, its observational nature and short follow-up period limit the assessment of long-term outcomes. Additionally, the number of patients with lung function data prior to therapy reinitiation was relatively small. Furthermore, because of the multicenter design of the study, and as certain parameters such as specific biomarkers, advanced imaging assessments, and treatment adherence were not incorporated into the initial study design, these evaluations were not performed and thus could not be included in the analysis. This may limit the interpretation of long-term structural and functional outcomes. However, as a real-world experience this multicenter study provides valuable insights and represents the first nationwide comparison of continuous and intermittent HEMT use.

## Conclusion

In conclusion, intermittent use of modulator therapy did not result in significantly worse short-term ppFEV1 and BMI outcomes compared to continuous use. However, interruptions in therapy may limit the cumulative benefits, leading to a smaller increase in lung function when compared to continuously treated patients. Studies on ETI in patients with cystic fibrosis have primarily focused on continuous use, and there are no planned studies investigating intermittent therapy. While intermittent use arising from necessity can provide real-world experience, it does not offer robust theoretical evidence. Insights from other chronic diseases suggest that intermittent treatment may increase the risk of resistance, reduce adherence, and potentially diminish long-term treatment efficacy. Given that our study represents a short-term observation, it does not allow for predictions regarding long-term outcomes. Furthermore, conducting a study on intermittent use in the current context would not be ethical. Although our unique real-world experience suggests that short-term deterioration in the care of CF patients is not significant, continuous use of therapy is nonetheless strongly recommended. Therefore, ensuring continuous access to HEMT in middle- and low-income countries should be prioritized to improve clinical outcomes and reduce global health disparities.

## Data Availability

The raw data supporting the conclusions of this article will be made available by the authors, without undue reservation.
